# Generation of orbital angular momentum modes via holographic leaky-wave metasurfaces

**DOI:** 10.1038/s41598-020-64278-9

**Published:** 2020-04-30

**Authors:** Homayoon Oraizi, Hedieh Emamian

**Affiliations:** School of Electrical Engineering, Iran University of Science and Technology, 1684613114 Tehran, Iran

**Keywords:** Engineering, Electrical and electronic engineering

## Abstract

Recently, electromagnetic (EM) waves carrying orbital angular momentum (OAM) has received considerable attention in increasingly many different realms, such as communication systems, super-resolution imaging, optical communications and quantum state manipulation. In this paper, two different kinds of two dimensional (2-D) holographic leaky-wave metasurfaces with a single OAM mode at a single frequency (18 GHz) are introduced through designs and experiments. The classic leaky-wave and a microwave holography theorem are combined to construct the holographic leaky-wave metasurfaces. The leaky wave metasurfaces-based holographic concept are implemented with isotropic artificial surface impedances and made of hexagonal metallic patches. By varying the size of the metallic patches, the effective impedances may be realized. The monopole launchers are utilized for the excitation of TM surface mode, whereby their wave functions can be approximated by the Hankel function of the second kind. The objective waves represented by the desired beams carrying different orbital angular momentum modes. Electromagnetic full-wave simulations and experimental measurements have been performed to substantiate the proposed method.

## Introduction

Wireless communication systems have gradually used innovative methods to meet the requirements of increased channel capacity and boost the transmission data-rate to develop communication applications. Conventional communication methods have faced the barriers of radio spectrum efficiency and the channel capacity expansion. One recently way to achieve higher capacities^1^ is to spatially multiplex multiple orthogonal modes using a single transmitter/receiver aperture pair, in which each mode carries an independent data stream. Orthogonality assures that the modes can be efficiently multiplexed at the transmitter, spatially co-propagated, and are demultiplexed at the receiver with minimal modal crosstalk^2^. As a new degree of freedom, due to the mutual orthogonality among eigenstates of the orbital angular momentum, scholars have conducted several versatile researches on the orbital angular momentum (OAM). Since then, beams carrying OAM inspires a new thinking in many diverse applications ranging from classical and quantum communication to optical manipulation^3–5^.

Similar to light beams, it is well-known that electromagnetic (EM) waves can carry both linear momentum (LM) and angular momentum (AM). The linear momentum of EM waves is associated with the Poynting’s vector. The angular momentum constitutes spin angular momentum (SAM) and orbital angular momentum (OAM)^6^ in the paraxial regime. The SAM defines the spin feature of photon and is associated with the polarization of the light and modes of EM waves. In the optical regime, it has only two value of $\pm h$ (Plank’s constant divided by 2π) per photon. In electromagnetics, three possible polarization modes may be attributed to the electromagnetic beam, namely the left- and right-circularly polarized modes and linearly polarized mode. These modes merely impose channel limitation in communication systems. On the other hand, OAM defines the orbital rotation of photon. Each photon has an OAM of $\,\pm lh$, where $\,l$ is known as a topological charge or OAM index, which can be any integer ranging from $-\infty \,$to $\infty$.

In 1992^7^, Allen *et al*. first demonstrated that light beams with an azimuthal phase distribution $\exp (il\varphi )$ carries an angular momentum independent of the polarization photon state, where $\varphi \,$is the azimuthal angle around the propagation axis and $l$ is the topological charge, which represents the number of twists of the wavefront. Before that, Soskin *et al*. created a structured light with helical wavefront by using forked gratings^8^. It has been used to demonstrate high-capacity optical and radio communication channels by employing multiple OAM states into the transmitted EM wave on the light. In 2007 Bo Thide introduced the OAM concept from an optical frequency to a radio band by using circular array antennas with a gradient phase difference^9^.

At present, various methods for generating EM OAM waves have been presented. In optics, computer-generated holograms (CGH)^10^, spiral phase plates (SPP)^11^, spatial light modulators (SML)^12^ and Q-plate have been generated by the spin-orbit coupling in inhomogeneous media^13^. At radio domain, circular phase array antennas^14,15^, dielectric resonator antennas^16^, reflect and transmit arrays^17,18^ and metasurfaces^19–21^ have been designed. Although there are various designs in different configurations, the design principles for OAM generation lead to one universal rule: the introduction of the azimuthal phase term $\exp (il\varphi )$ to EM waves.

By comparison with the conventional methods, owing to the extreme controls of EM waves, (namely low loss, low profile, low cost, and high performance), it has been proved that metasurfaces are more convenient to create versatile radiation beams^22^. Generally, metasurfaces, which are the planar version of bulky 3D metamaterials are divided into two categories including periodic and quasi-periodic structures. The first category is widely used in frequency selective structures^23^, polarization convertors^24^ and polarization rotators^25^. Meantime, much attention has been focused on the quasi-periodic structures, by reason of providing more design freedom to perform inhomogeneous surfaces. Various prototypes of radiators have been proposed for OAM generation, such as golden nanorods in^26^, L-shaped gold nanoantennas^27^, artificial perfect-electric and perfect-magnetic conductors anisotropic metasurface^28^, ultra-thin complementary metasurface aimed for double-layer complementary metasurface for OAM generation with high efficiency and metasurface Fork gratings^29^.

It is necessary to provide a more appropriate and efficient design procedure. The metasurfaces shaped by artificial impedance surfaces have many applications in designing leaky-wave antennas^30^. Surface wave leakage manipulation is one of the important applications of metasurface^31^. Wave leakage can be made by perturbation in the propagation medium. The great advantage of the leaky-wave systems is controlling the amplitude and propagation direction of the leaky waves^32^. Combining with the microwave holography principle, the desired wave can be generated by exciting the hologram interference of the reference wave and object wave in any desired direction^33^. The physical equivalence between the leaky waves and holographic antennas based on the Oliner’s method was given in^34^. The holography principle in optics was first introduced by Gabor^35^. Checcacci^36^ then utilized it at microwave frequencies. Since it has high potentials in various applications, research has been undertaken extensively on this theory. Sievenpiper^37^ proposed the hologram leaky wave for pattern synthetization. Periodic ground patches supporting $T{M}_{0}$ surface wave are used to realize this concept. The new degree of freedom, which is the possibility of polarization manipulation has been added to hologram designs using anisotropic unit-cells^38^.

In this paper, we propose two different kinds of holographic leaky-wave metasurfaces to generate OAM modes with $l=2$ and $l=3$ at 18 GHz. The metasurfaces comprise of hexagonal patches on the grounded dielectric. We use the monopole antenna to excite the interferogram, which is the recorded interference of reference and object waves for the generation of the desired OAM beams.

## The principle of holography for designing the leaky-wave metasurface

The evolution of the holography concept, which was indicated as a two-step imaging process, opened a new vision into wave phenomena and leading to development of a new class of antenna named holographic antenna^39^. It is important to explain the two-step imaging process, including the hologram formation and wave front reconstruction steps. Based on the microwave holographic theory, the reference wave excites the hologram and is consistent with the wave scattered from the object, which is called object wave. The intervention of the reference wave and object wave is called the interferogram pattern^40^. The desired radiation waves can be generated by exciting the hologram interference (generated by the reference wave and object wave). holographic leaky wave metasurfaces are assembled of quasi-periodic surface impedance. If (*ψ*_*ref*_) and (*ψ*_*obj*_) define the reference wave and object waves, respectively, the surface impedance realizing the object wave is given by:

1 $${Z}_{s}=j{X}_{0}{\eta }_{0}(1+M\times  {\mathcal R} \{{\psi }_{ref}{\psi }_{obj}^{\ast }\})$$

where ${X}_{0}$ is the normalized impedance with respect to the free space intrinsic impedance (${\eta }_{0})$, $M$ is the modulation depth coefficient, ${\mathcal R}$ defines the real part of the $({\psi }_{ref}{\psi }_{obj}^{\ast })$ and “*” denotes the complex conjugation operation. The reference wave may be selected as the surface or space wave. Here, for our proposed metasurfaces, the reference waves are defined as cylindrical waves in the form of Hankel function of the second kind:

2 $${\psi }_{ref}=\alpha {H}_{0}^{(2)}(\beta \sqrt{{x}^{2}+{y}^{2}})$$

where $\beta$ is the phase constant for the $T{M}_{0}$ surface mode and is obtained as

3 $$\beta ={k}_{0}\sqrt{1+{X}_{0}^{2}}$$

Observe that $$\beta \,$$is an approximation of the wave number which is applied for the object wave calculation and derivation of the associated surface impedance. The modulation factor ($$M)$$ should be small value to justify the approximated value of $$\beta $$. Otherwise, if the modulation depth is not small, the phase constant ($$\beta )$$ may be obtained by the dispersion relation. The desired object wave (radiation wave) in this work in a specific direction is represented as:

4 $${\psi }_{obj}={e}^{-j(kxsin{\theta }_{0}cos{\varphi }_{0}+kysin{\theta }_{0}sin{\varphi }_{0}+l\phi )}$$

where ${\theta }_{0}\,$and *φ*_0_ are the main lobe direction angles in the XZ and XY planes, respectively. $l$ is the topological charge and defines the number of twists.

**Figure 1:**
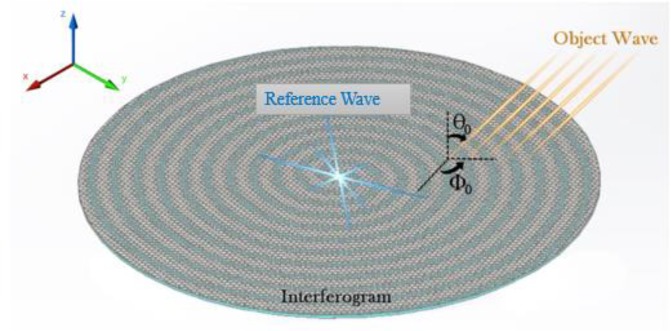
The schematic views of the cylindrical reference wave, object wave and interferogram.

The schematics in Fig. 1 demonstrate the hologram principle and leaky wave theory to design the holographic leaky-wave metasurface. It illustrates that exciting the interferogram or holographic surface impedance by the reference wave, the object wave or desired radiation wave is generated.

## Higher-ordered bessel beam and orbital angular momentum

Non-diffracting Bessel beams are a set of solutions^41^ of the free space Helmholtz equation and have transverse intensity profiles that can be described by the Bessel function of the first kind. They have several features, such as non-diffraction, self-reconstruction, and even providing optical pulling forces. The scalar form of Bessel beams wave-function can be introduced as:

5 $$E(r,\varphi ,z)=A.\exp (i{k}_{z}z).{J}_{n}({k}_{r}r).\exp (\pm jn\varphi )$$

where $\,A$ is the amplitude $,\,{k}_{z}\,$and $\,{k}_{r}$ are the corresponding transverse and longitudinal propagation constants that satisfy the equation $\sqrt{{k}_{z}^{2}+{k}_{r}^{2}}=k=2\pi /\lambda {\prime}$. It can be derived from Eq. (5) that the transverse intensity profiles of Bessel beams are independent of $z$ coordinate which gives rise to their non-diffracting characteristic. Due to that OAM is structured beam with phase term $\exp (il\varphi )$ and is related to vorticity and phase singularity it also shows that any higher-ordered Bessel beam $(n\ne 0)$ must carry an orbital angular momentum and have zero intensity along the $z$ axis at $r=0$ because of the phase singularity resulting from $\exp (\pm jn\varphi )$ term.

The general form of the aperture field for the Bessel beams which carry an orbital angular momentum with the mode of $(n+1)$ can be defined as:

6 $$\overrightarrow{E}(r,\varphi ,z)=({E}_{r}\hat{r}+{E}_{\varphi }\hat{\varphi }+{E}_{z}\hat{z}).{e}^{-j{k}_{z}z}{e}^{-j(n+1)\varphi }$$

${E}_{r}$, ${E}_{\varphi }$, and ${E}_{z}$ components are:

7 $$(\begin{array}{c}(-\,{C}_{TE}\frac{Jn({k}_{r}r)+Jn+2({k}_{r}r)}{2}+i.{C}_{TM}{e}^{in}\sqrt{1-N{A}^{2}}\frac{Jn({k}_{r}r)+Jn+2({k}_{r}r)}{2})\mathop{r}\limits^{\frown {}}\\ -({C}_{TM}{e}^{in}\sqrt{1-N{A}^{2}}\frac{Jn({k}_{r}r)+Jn+2({k}_{r}r)}{2}+i.{C}_{TE}\frac{Jn({k}_{r}r)+Jn+2({k}_{r}r)}{2})\mathop{\phi }\limits^{\frown {}}\\ NA.{C}_{TM}{e}^{in}\,{J}_{n+1}({k}_{r}r)\mathop{z}\limits^{\frown {}}\end{array})$$

where ${C}_{TM}$ and $\,{C}_{TE}\,$are complex numbers associated with the constituent transverse magnetic and transverse electric waves of Bessel beams; $\eta$ is the phase difference between ${C}_{TM}\,$and $\,{C}_{TE}.$ The coefficient of $NA$ shows the contribution of $z$ component in the near field. For high NA Bessel beams, the electric field of the $z$ component results from TM waves: the higher the NA, the higher the contribution the TM waves make to ${E}_{z}$. The ${E}_{r}$ and ${E}_{\varphi }$ components mainly result from TE wave contribution, as the term $\sqrt{1-N{A}^{2}}$ is relatively small for the high NA case.

Now consider that the value of ${k}_{r}r$ is large enough, so the argument of Bessel function can be approximated as:

8 $${J}_{n}({k}_{r}r)\approx \sqrt{\frac{2}{\pi {k}_{r}r}}\,\cos \left({k}_{r}r-\frac{\pi }{4}-\frac{n\pi }{2}\right)$$

So ${J}_{n}({k}_{r}r)$ and ${J}_{n+2}({k}_{r}r)$ cancel each other due to a $\pi$ phase difference, and the $\varphi$ component of the aperture will be zero. These coefficients also have direct contributions in the far field pattern, and their effects will be canceled in the far field. As a result, in the far field approximation, by using isotropic unit-cells we can generate OAM modes.

## Analysis of monopole launcher as a reference wave

Choosing the proper reference wave is the crucial step of the design procedure of modulated metasurfaces. The monopole launcher on a grounded dielectric medium is selected for the excitation of $T{M}_{0}$ surface waves. The electric and magnetic fields of the monopole launcher can be defined as^42^:

9 $${\overrightarrow{E}}_{sw}=\left[j{X}_{s}{J}_{sw}{H}_{1}^{(2)}({\beta }_{sw}\,\rho )\hat{\rho }+{J}_{sw}\frac{{\beta }_{sw}\,{\eta }_{0}}{{k}_{0}}{H}_{0}^{(2)}({\beta }_{sw}\,\rho )\hat{z}\right]{e}^{-{\alpha }_{z}z}$$

10 $${\overrightarrow{H}}_{sw}=-\,{J}_{sw}{H}_{1}^{(2)}({\beta }_{sw}\,\rho ){e}^{-{\alpha }_{z}z}\hat{\varphi }$$

where ${\beta }_{sw}$ and ${\alpha }_{z}$ are the radial propagation constant and the attenuation constant in the $z\,$direction, respectively. The radial propagation constant ${\beta }_{sw} > k$ obeys the dispersion equation:

11 $${\beta }_{sw}=k\sqrt{1+\overline{{X}_{s}^{2}}/{{\eta }_{0}}^{2}}$$

12 $${\alpha }_{z}=\sqrt{{\beta }_{sw}^{2}-{k}^{2}}=\frac{k}{{\eta }_{0}}\overline{{X}_{s}}$$

${X}_{s}$ is the positive surface reactance of the grounded dielectric and TM-SW can be excited only if the reactance is positive.

It can be verified that the monopole launcher with moment ${I}_{0}\Delta {\rm{z}}$ have a relation with complex amplitude $\,{J}_{sw}$ through:

13 $${J}_{sw}=-\,{I}_{0}\Delta {\rm{z}}\frac{{k}_{0}{\beta }_{sw}{X}_{s}}{2{\eta }_{0}}$$

The power transferred by the electric and magnetic fields is:

14 $$\begin{array}{c}{\overrightarrow{P}}_{SW}={\overrightarrow{E}}_{SW}\times {\overrightarrow{H}}_{SW}\ast =-\,jX{|{J}_{SW}|}^{2}{|{H}_{1}^{(2)}({\beta }_{SW}\rho )|}^{2}e-2{\alpha }_{z}z\mathop{z}\limits^{\frown {}}+\frac{{\eta }_{0}{\beta }_{SW}}{{k}_{0}}{|{J}_{SW}|}^{2}{H}_{0}^{(2)}\\ ({\beta }_{SW}\rho ){H}_{1}^{(2)}\ast ({\beta }_{SW}\rho )e-2{\alpha }_{z}{z}^{z}\hat{\rho }\sqrt{{b}^{2}-4ac}\end{array}$$

The Poynting’s vector along the $\rho$ and $z$ directions shows the coupling of waves to the dielectric medium. The power density cannot leave the dielectric^43^, and the propagation only occurs along the $\rho$ direction, because the $\hat{z}$ component of the Poynting’s vector is pure imaginary. The real part of the Poynting’s vector represents the propagation along the $\rho$ direction. Also, only this part has a contribution to the holography Eq. (1).

## Holographic design of leaky-wave metasurfaces for oam beams

**Figure 2:**
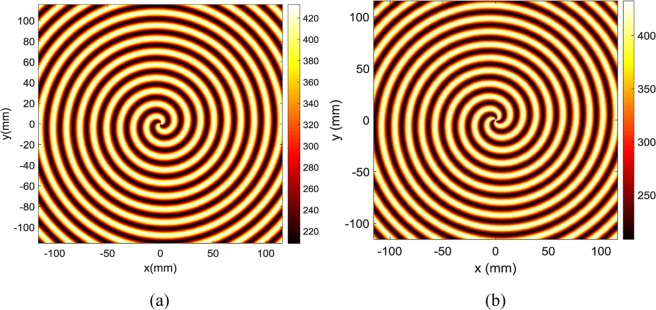
(**a**) The interference pattern of the reference wave and the object wave in direction ${\theta }_{0}$= 0 and *φ*_0_ = 0 for OAM mode with topological charge 2. **(b)** The interference pattern of the reference wave and the object wave in direction ${\theta }_{0}$= 0 and *φ*_0_ = 0 for OAM mode with topological charge 3.

Figure 2a,b show the 2-D holographic interference patterns of cylindrical surface wave and desired OAM beams with the topological charge of 2 and 3, respectively. The interference patterns are signified to generate desired object waves at the direction of ${\theta }_{0}$ = 0 and *φ*_0_ = 0. The color bar on the right shows the imaginary value of the patterns for the sample from minimum to maximum. These interferograms are extractions of the holographic equation of surface impedance (1) and the MATLAB software. For the constitution of surface impedances, it is all important that the dimensions of unit-cell be small relative to the wavelength. For the implementation of surface impedances, the grounded unit-cell patches (in the shape of square, circular, and hexagonal) are suitable.

**Figure 3:**
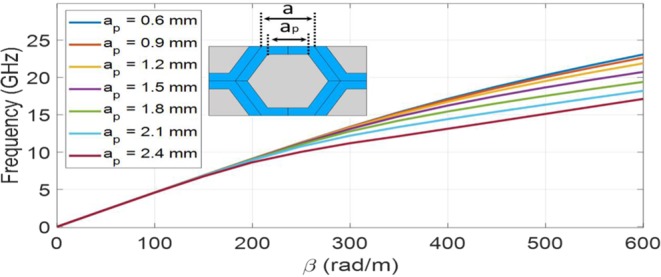
Geometry of the proposed unit cell and the dispersion diagram of the proposed unit-cell in terms of different values of *a*_*p*_.

Considering the dispersion isotropy^44^, the hexagonal unit-cell is more well-behaved. Figure 3. represents the geometry of proposed unit-cell and its dispersion diagram in terms of different values of *a*_*p*_. It is notable that $\beta$ have direct relation with surface impedance. The variations of the dispersion diagram represent the variations of surface impedance, moreover with changing the values of *a*_*p*_, the value of surface impedance changes. The value of *a* in Fig. 3 is 2.6 mm. The proposed method in^45^ is adopted to calculate the surface impedance.

**Figure 4:**
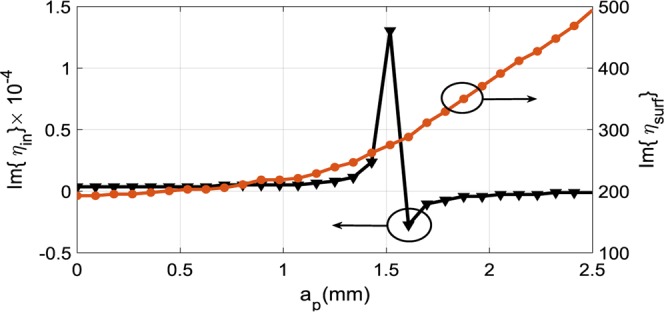
The curves of variations of imaginary parts of *η*_*in*_ and *η*_*surf*_ at frequency 18 GHz as a function of the length of the hexagonal edge (*a*_*p*_).

**Figure 5:**
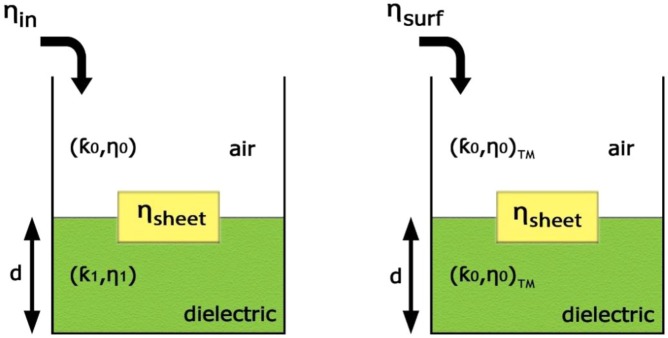
The equivalent transmission line of the proposed unit cell. (**a**) Normal incident model; (**b**) TM mode model.

Figure 4 represents the curves of variations of imaginary parts of *η*_*in*_ and *η*_*surf*_ at frequency 18 GHz as a function of the length of the hexagonal edge (*a*_*p*_). Figure 5 represents the equivalent transmission line circuit for the normal incident wave and TM mode. The surface impedance can be obtained by the conflation of the two models of transmission line in Fig. 5.

The input impedance, which is related to the reflection coefficient can be attained by a full wave simulation, which is associated with the reflection coefficient as:

15 $${\eta }_{in}={\eta }_{0}\frac{1+\Gamma }{1-\Gamma }$$

We can obtain the surface impedance for the normal incidence (*η*_*sheet*_) which is shown in Fig. 5a with the aid of Eq. (16):

16 $$\frac{1}{{\eta }_{sheet}}=\frac{1}{{\eta }_{in}}-\frac{1}{j{\eta }_{1}\,\tan ({k}_{1}d)}$$

By using *η*_*sheet*_, the surface impedance of the incident TM mode (*η*_*surf*_) may be obtained from the circuit in Fig. 5b:

17 $$\frac{1}{{\eta }_{surf}}=\frac{1}{{\eta }_{sheet}}+\frac{1}{j\frac{{\eta }_{0}k{z}_{1}}{{k}_{0}{\varepsilon }_{r1}}\,\tan (k{x}_{1}d)}$$

where:

18 $${k}_{{x}_{1}}=\sqrt{{k}_{0}^{2}({\varepsilon }_{r}-1)+{\left(\frac{{\eta }_{surf}{k}_{0}}{{\eta }_{0}}\right)}^{2}}$$

As the interference patterns shown in Fig. 2a,b, two different types of holographic leaky-wave antennas are proposed for the generation of OAM modes with topological charges of $l=2$ and $l=3$ operating at single frequency of 18 GHz in the direction of ($\theta ={0}^{^\circ },\,\varphi ={0}^{^\circ })$, respectively. The substrate Rogers RO4003C is used in this work, which has a dielectric constant of ${\varepsilon }_{r}=3.55$, $\,tan\delta =0.0027$, and thickness of 1.524 mm. The surface impedance (comprised of different sizes of hexagonal patches) is designed to produce the desired object waves. The values of *M* and *X*_0_ are arbitrary. However, their values should be selected in such a way that the impedance variation lies in the range of values obtainable by the synthesized unit-cells. Therefore, they are selected as *X*_0_ = 0.85 and *M* = 0.35. When the incident electromagnetic waves (reference wave) passes through the modulated metasurfaces, the desired radiation of OAM beams are generated.

**Figure 6:**
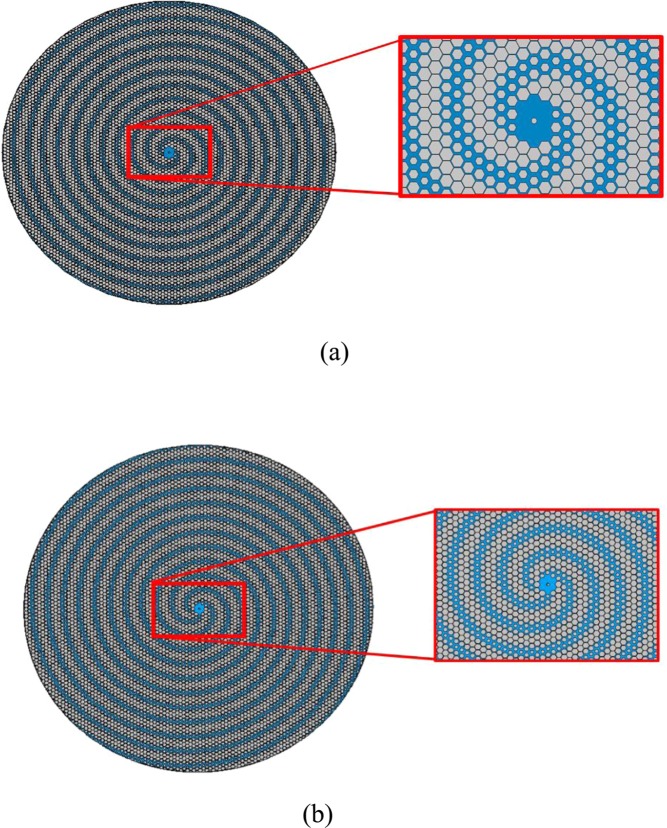
(**a**) The center of Holographic Leaky-wave metasurface for OAM mode $l=2$. (**b**) The center of Holographic Leaky-wave metasurface for OAM mode $l=3$.

Figure 6a,b depict the shapes of the centers of the metasurfaces. Due to that the desired object waves are different, the distinctive center of metasurfaces are observable according to the figures. Note that, the surface impedance depends on the incident and object waves.

## Determination of the aperture field approximation

The generalized form of the holographic theory is proposed for the synthesis of the anisotropic hologram using the aperture field approximation^46^. It is proposed in this paper that for the generation of the OAM beam of mode 1, the synthesis of anisotropic surface impedances is required by the aperture field approximation.

For the generation of OAM beams, the desired object wave is represented by ${\psi }_{obj}$ as an aperture electric field (${\overrightarrow{E}}_{a})$:

19 $${\psi }_{obj}={\overrightarrow{E}}_{a}={e}^{-j(\overrightarrow{k}.\overrightarrow{r})-jl\mathop{\varphi }\limits^{ \acute{} }}\check{\rho }$$

If the aperture field is known, the antenna far-field may be calculated by^47^:

20 $$\overrightarrow{E}(r,\theta ,\varphi )=\frac{jk{e}^{-jkr}}{2\pi r}({F}_{\theta }(\theta ,\varphi )\hat{\theta }+{F}_{\varphi }(\theta ,\varphi )\hat{\varphi })$$

where ${F}_{\theta }(\theta ,\varphi )$ and ${F}_{\varphi }(\theta ,\varphi )$ are the Fourier transforms of aperture field.

21 $${F}_{\theta }(\theta ,\varphi )={f}_{x}\,\cos \,\varphi +{f}_{y}\,\sin \,\varphi $$

22 $${F}_{\varphi }(\theta ,\varphi )=\,\cos \,\theta ({f}_{y}\,\cos \,\varphi -{f}_{x}\,\sin \,\varphi )$$

where ${f}_{x}$ and ${f}_{y}\,$are Fourier transforms of aperture field along the $x$ and $y$ axes, respectively obtained as:

23 $${f}_{x}({k}_{x},{k}_{y})=\int {E}_{ax}(\mathop{x}\limits^{ \acute{} },\mathop{y}\limits^{ \acute{} },\mathop{z}\limits^{ \acute{} }=0){e}^{j({k}_{x}\mathop{x}\limits^{ \acute{} }+{k}_{y}\mathop{y)}\limits^{ \acute{} }}d\mathop{x}\limits^{ \acute{} }d\mathop{y}\limits^{ \acute{} }$$

24 $${f}_{y}({k}_{x},{k}_{y})=\int {E}_{ay}(\mathop{x}\limits^{ \acute{} },\mathop{y}\limits^{ \acute{} },\mathop{z}\limits^{ \acute{} }=0){e}^{j({k}_{x}\mathop{x}\limits^{ \acute{} }+{k}_{y}\mathop{y)}\limits^{ \acute{} }}d\mathop{x}\limits^{ \acute{} }d\mathop{y\,}\limits^{ \acute{} }\,$$

where *E*_*ax*_ and *E*_*ay*_ are the aperture field components in the Cartesian coordinate axes, which may be obtained by decomposing $\,\check{\rho }=cos\mathop{\varphi }\limits^{ \acute{} }\check{x}+sin\mathop{\varphi }\limits^{ \acute{} }\,\check{y}$, so:

25 $${\overrightarrow{E}}_{a}={e}^{-j(\overrightarrow{k}.\overrightarrow{r})-jl\mathop{\varphi }\limits^{ \acute{} }}\check{\rho }={e}^{-j(\overrightarrow{k}.\overrightarrow{r})-jl\mathop{\varphi }\limits^{ \acute{} }}(cos\mathop{\varphi }\limits^{ \acute{} }\check{x}+\,sin\mathop{\varphi }\limits^{ \acute{} }\,\check{y})$$

Substitute $\,\mathop{x}\limits^{ \acute{} }=\,\mathop{\rho \,}\limits^{ \acute{} }cos\mathop{\varphi \,}\limits^{ \acute{} }$, $\mathop{y}\limits^{ \acute{} }=\,\mathop{\rho \,}\limits^{ \acute{} }\,sin\mathop{\varphi ,}\limits^{ \acute{} }\,{k}_{x}={k}_{0}sin\theta \,\cos \,\varphi$ and ${k}_{y}={k}_{0}sin\theta sin\varphi$ into Eq. (25) to get:

26 $${E}_{ax}={e}^{-j{k}_{0}\mathop{\rho }\limits^{ \acute{} }sin{\theta }_{0}\cos (\mathop{\varphi }\limits^{ \acute{} }-{\varphi }_{0})-jl\mathop{\varphi }\limits^{ \acute{} }}cos\mathop{\varphi }\limits^{ \acute{} }\check{x}$$

27 $${E}_{ay}={e}^{-j{k}_{0}\mathop{\rho }\limits^{ \acute{} }sin{\theta }_{0}\cos (\mathop{\varphi }\limits^{ \acute{} }-{\varphi }_{0})-jl\mathop{\varphi }\limits^{ \acute{} }}sin\mathop{\varphi }\limits^{ \acute{} }\,\check{y}$$

Also,

28 $${k}_{x}\mathop{x}\limits^{ \acute{} }+{k}_{y}\mathop{y\,=\,}\limits^{ \acute{} }{k}_{0}sin\theta \,\cos \,\varphi \mathop{\rho \,}\limits^{ \acute{} }cos\mathop{\varphi }\limits^{ \acute{} }+{k}_{0}sin\theta sin\varphi \mathop{\rho \,}\limits^{ \acute{} }\,sin\mathop{\varphi }\limits^{ \acute{} }=\,{k}_{0}\mathop{\rho \,}\limits^{ \acute{} }sin\theta \,\cos (\mathop{\varphi }\limits^{ \acute{} }-\,\varphi )$$

By inserting Eqs. (26), (27) and (28) into Eq. (23) and (24):

29 $${f}_{x}={\int }_{0}^{2\pi }{\int }_{0}^{a}\cos \,\varphi {\prime} {e}^{jk}0\rho {\prime} (\sin \,\theta \,\cos (\varphi {\prime} -\varphi )-\,\sin \,{\theta }_{0}\,\cos (\varphi {\prime} -\varphi )).{e}^{-jl\varphi }\rho {\prime} d\rho {\prime} d\varphi {\prime} $$

30 $${f}_{y}={\int }_{0}^{2\pi }{\int }_{0}^{a}\sin \,\varphi {\prime} {e}^{jk}0\rho {\prime} (\sin \,\theta \,\cos (\varphi {\prime} -\varphi )-\,\sin \,{\theta }_{0}\,\cos (\varphi {\prime} -\varphi )).{e}^{-jl\varphi }\rho {\prime} d\rho {\prime} d\varphi {\prime} $$

By substituting Eqs. (29) and (30) into Eqs. (21) and (22):

31 $${F}_{\theta }=\,\iint \,\cos (\mathop{\varphi }\limits^{ \acute{} }-\,\varphi )\,{e}^{j{k}_{0}\mathop{\rho }\limits^{ \acute{} }(sin\theta \cos (\mathop{\varphi }\limits^{ \acute{} }-\varphi )-sin{\theta }_{0}\cos (\mathop{\varphi }\limits^{ \acute{} }-{\varphi }_{0}))}.\,{e}^{-jl\mathop{\varphi }\limits^{ \acute{} }}\mathop{\rho \,}\limits^{ \acute{} }d\mathop{\rho \,}\limits^{ \acute{} }d\mathop{\varphi }\limits^{ \acute{} }$$

32 $${F}_{\varphi }=\iint sin(\mathop{\varphi }\limits^{ \acute{} }-\,\varphi )\,{e}^{j{k}_{0}\mathop{\rho }\limits^{ \acute{} }(sin\theta \cos (\mathop{\varphi }\limits^{ \acute{} }-\varphi )-sin{\theta }_{0}\cos (\mathop{\varphi }\limits^{ \acute{} }-{\varphi }_{0}))}.\,{e}^{-jl\mathop{\varphi }\limits^{ \acute{} }}\mathop{\rho \,}\limits^{ \acute{} }d\mathop{\rho \,}\limits^{ \acute{} }d\mathop{\varphi }\limits^{ \acute{} }$$

Consequently, for the isotropic structure with $\,{\rm{l}}=1$, the polarization is circular since ${F}_{\varphi }=(-j){F}_{\theta }$. However, due to ${F}_{\varphi }\& \,{F}_{\theta }\,\ne 0$, there is no null in the pattern. Note that one of the main characteristics of the OAM beams is the existence of a null and phase singularity in the pattern. Therefore, for the generation of topological charge $l=1$, we need to synthesize an anisotropic surface impedance.

## Experimental results

**Figure 7:**
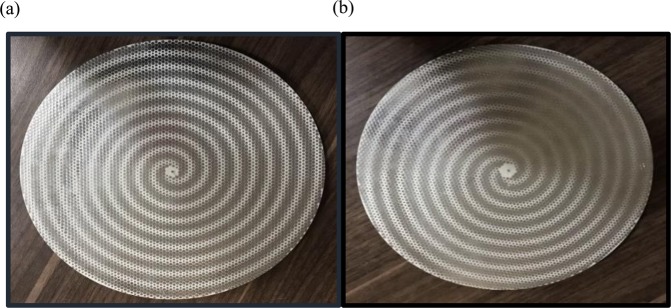
(**a**) The fabricated holographic leaky-wave metasurfaces with topological charges of OAM of $l=2$. (**b**) The fabricated holographic leaky-wave metasurfaces with topological charge of OAM of $l=3$.

**Figure 8:**
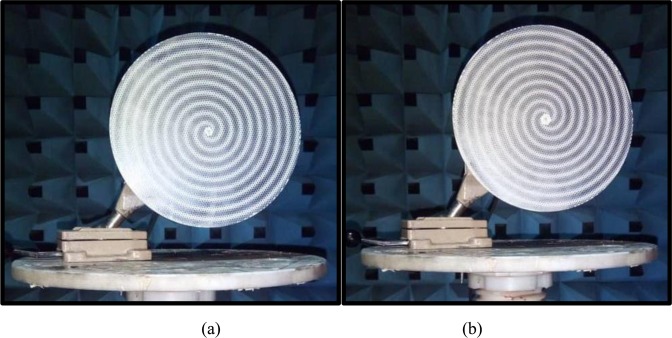
Experimental set-up of the proposed metasurfaces with topological charge of (**a**) $l=2\,$ (**b**) $\,l=3$.

**Figure 9:**
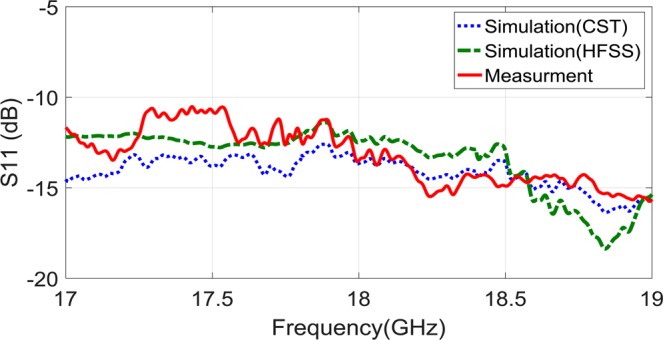
The simulation and measurement results for the holographic leaky-wave metasurfaces with topological charges of OAM of $l=2$.

**Figure 10:**
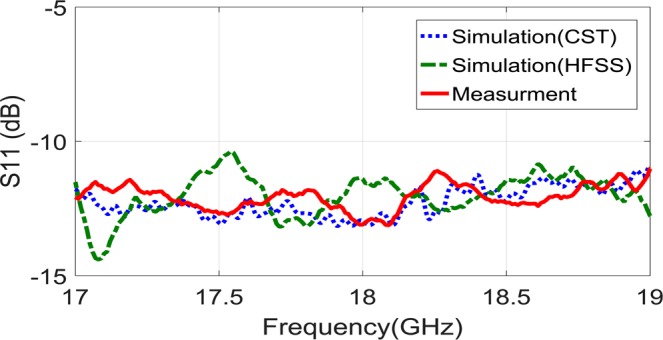
The simulation and measurement results for the holographic leaky-wave metasurfaces with topological charges of OAM of $l=3$.

As Fig. 7a,b demonstrate, the proposed holographic leaky-wave metasurfaces with different OAM modes of $$l=2$$ and $$l=3$$ are fabricated by using PCB technology. To vindicate our approach, the fabricated metasurfaces are measured in an anechoic chamber as shown in Fig. 8a,b. The aperture size of both metasurfaces are similar as D = 117.75 mm. Each element has a hexagonal lattice with a period of 2.6 mm. It is mentionable that, the distance between the unit-cells are a pivotal value for reducing the return loss ($${S}_{11})$$. Also, in order to avoid spurious radiation, the hexagonal patches around the launcher are removed. Furthermore, each metasurfaces have the identical circular apertures located at the center of them. For generating reference waves, exciting the metasurfaces and impedance matching, it is essential to solder the SMA connectors behind the structures and pass them through the circular apertures. The length of the monopole was selected to be 6.724 mm (0.4 $$\lambda $$ at 18 GHz). Also, the simulations are done through CST and HFSS software by the Computer System characteristics of: CPU Corei7 6800 K/RAM: 64 GByte/Num. of Thread: 12/Num. of Cores: 6. The Figs. 9 and 10 show the simulation and measured reflection coefficients. The figures show that the simulated and measured values of S-parameters $${S}_{11}$$ are below −10 dB ranges from 17 GHz to 19 GHz.

**Figure 11:**
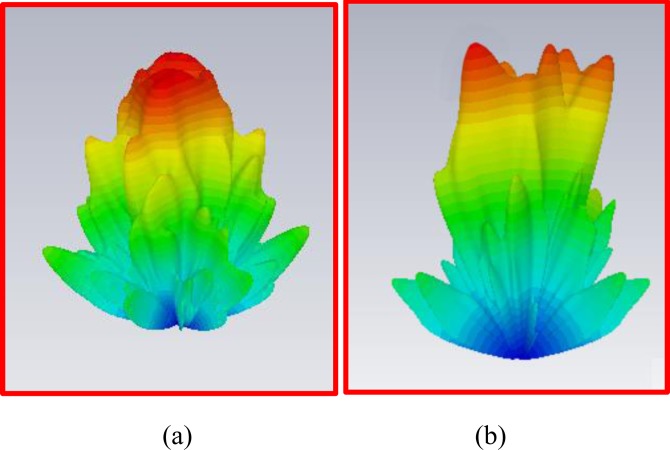
3D far-field radiation patterns. (**a**) 3D far-field radiation pattern for OAM mode with $l=2$. (**b**) 3D far-field radiation pattern for OAM mode with $l=3$.

**Figure 12:**
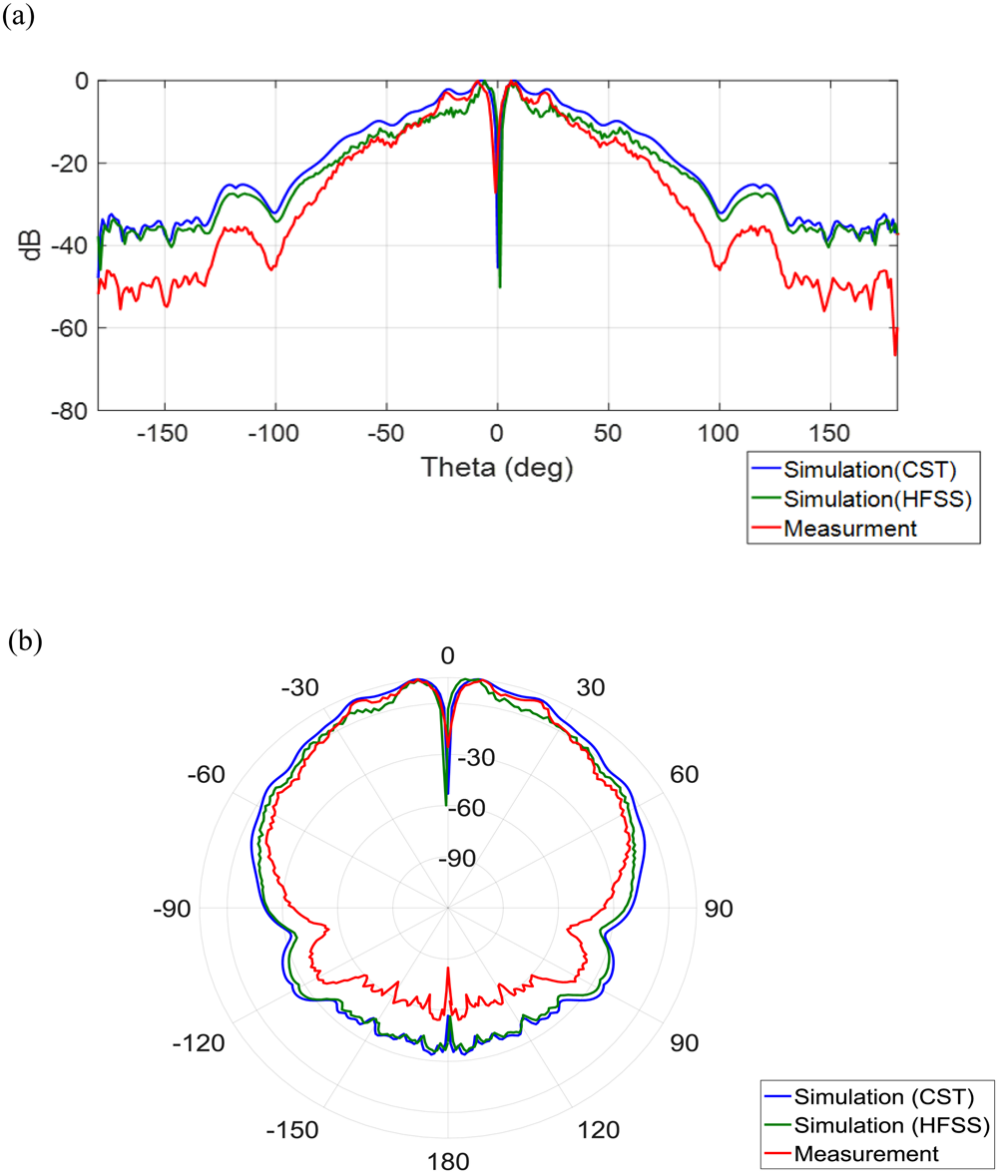
Simulated and measured patterns. (**a**) Simulated and measured Cartesian patterns for OAM mode with $l=2.$ (**b**) Simulated and measured polar patterns for OAM mode with $l=2$.

**Figure 13:**
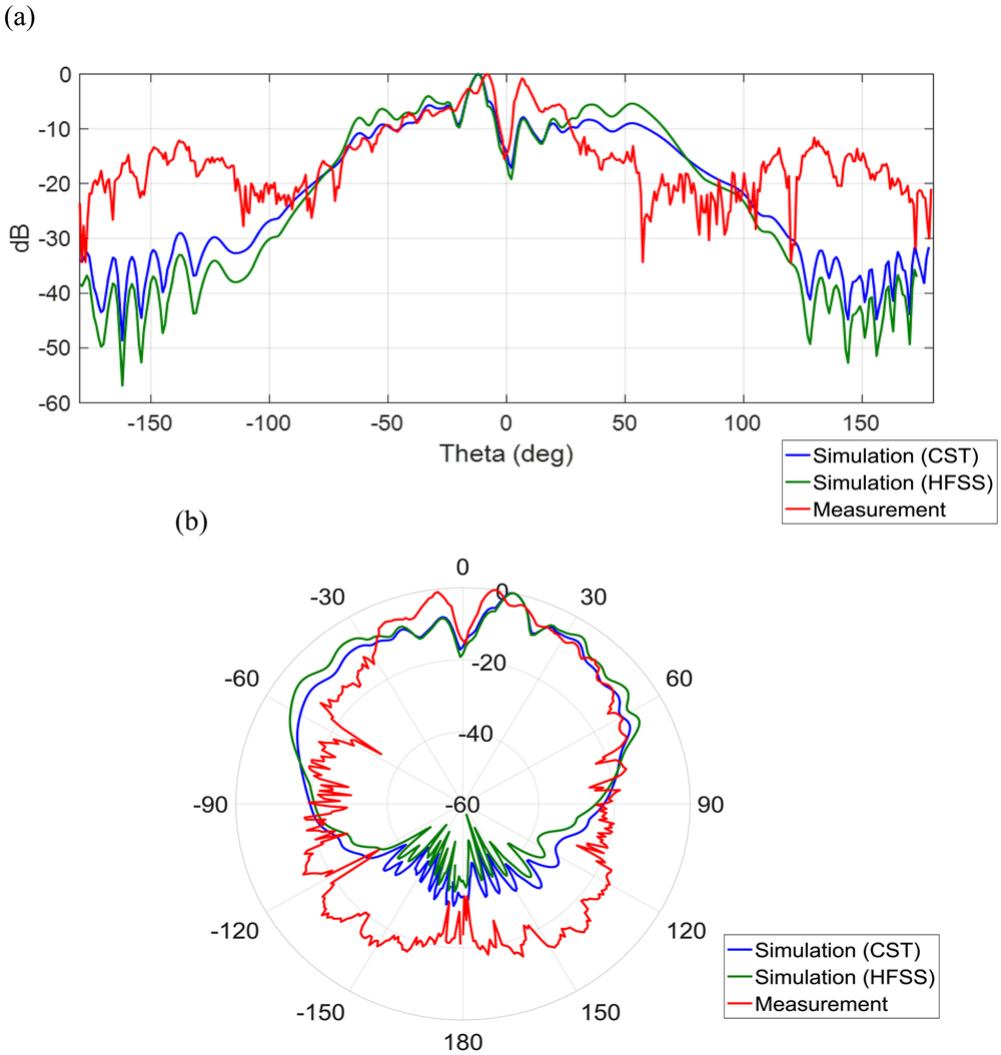
Simulated and Measured patterns. (**a**) Simulated and measured Cartesian patterns for OAM mode with $l=3$. (**b**) Simulated and measurements polar patterns for OAM mode with $l=3$.

To study the far-field radiation behavior, a standard horn antenna is located 6.5 m away from the metasurfaces. Figure 11 show the simulated 3D far-field radiation pattern. Figures 12 and 13 show the simulated and measured Cartesian and polar patterns for the OAM modes. As the polar and Cartesian patterns show, there are zero depths in the desired direction, which are in accordance with the properties and features of EM OAM beams.

According to Fig. 13, the reason for the discrepancy between the simulation results and measurement data of the far-field pattern for the OAM mode 3, could be attributed to the imprecision of fabrication processes of metasurfaces in our laboratory. Also, the measurement equipment may not have been calibrated properly for the higher order OAM modes. Note that the resolution and dimensions of the unit-cells are the limitations for synthesizing the impedance surface pattern for generating the higher order OAM modes. Also, due to the mentioned difficulties and limitations the far-filed pattern of mode 3 is somewhat not symmetrical.

**Figure 14:**
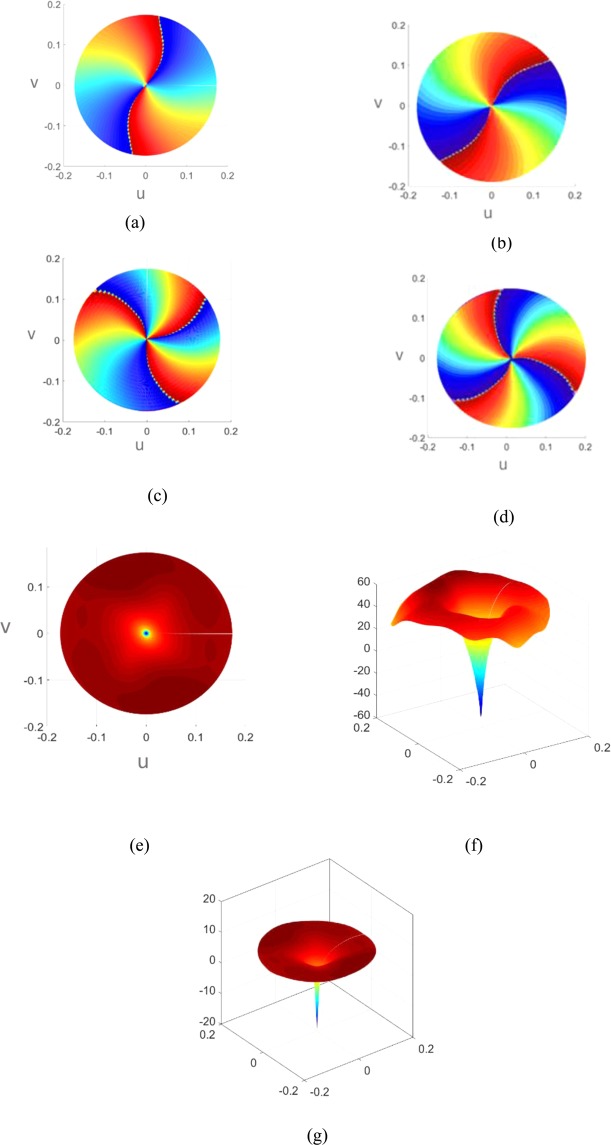
(**a**,**b**) The demonstration of CST and HFSS Simulation Phi-phase pattern of the holographic leaky-wave metasurfaces with topological charges of OAM of $l=2\,$,respectively (**c**,**d**)) The demonstration of CST and HFSS Simulation Phi-phase pattern of the holographic leaky-wave metasurfaces with topological charges of OAM of $l=3$, respectively (**e**) The demonstration of CST simulation of intensity pattern of the holographic leaky-wave metasurfaces with topological charges of OAM of $l=2$ and $l=3.$ (**f**,**g**) The demonstration of HFSS simulation of intensity pattern of the holographic leaky-wave metasurfaces with topological charges of OAM of $l=2$ and $l=3,$ respectively.

To verify the unique properties and features of the OAM beams, the intensity and phi-phase pattern of metasurfaces are obtained with simulations through CST and HFSS. By exporting the far-field data from the CST and HFSS and importing them to MATLAB, the data for Fig. 14 are extracted and drawn. For the figures depicting the field strength, the plots of the magnitude and phase of the far-field are drawn. Note that the vertical and the horizontal axes are in terms of u = $sin\theta cos\varphi$ and $v=\,sin\theta sin\varphi$, respectively.

The Fig. 14 show the phi-phase and transverse intensity pattern of the OAM beams. Observe that a clear doughnut-shaped intensity pattern appears with a singularity in the center, and a spiral-shaped phase figures show that our proposed metasurfaces significantly generate OAM modes. Also, Fig. 14f,g represent the depth of the nulls in the intensity patterns for modes of $l=2$ and $l=3$, where the depth of the nulls are compliance with the Figs. 12a and 13a.

## Conclusion

We have successfully demonstrated two different leaky-wave metasurfaces for the generation of OAM beams. The leaky wave theory and the microwave holography method were combined to attain the interference pattern. The proposed holographic leaky-wave metasurfaces comprise of hexagonal patches which have variation in their sizes and dimensions. The patches are suitable for any direction of incident waves. We have also performed computer simulations and conducted measurements for the fabricated metasurfaces. The simulation results were in good agreement with those of experimental measurements, which strongly demonstrated the excellent performance of our design. Due to their ultralow profile, low cost, compact structure and small size and easy fabrication and integration, these metasurfaces are expected to be an effective solution for the OAM mode generations.

## References

[1] Demers, F., Yanikomeroglu, H. & St-Hilaire, M. A survey of opportunities for free space optics in next generation cellular networks. Paper presented at the Ninth Annual Communication Networks and Services Research Conference (CNSR), Ottawa ON, Canada (doi: 10.1109/CNSR.2011.38) (2011, May 2–5).

[2] Ahmed N (2016). Mode-division-multiplexing of multiple Bessel-Gaussian beams carrying orbital-angular-momentum for obstruction-tolerant free-space optical and millimetre-wave communication links. Scientific reports.

[3] Grier DG (2003). A revolution in optical manipulation. Nature.

[4] Leach J (2010). Quantum correlations in optical angle-orbital angular momentum variables. Science.

[5] Wang J (2012). Terabit free-space data transmission employing orbital angular momentum multiplexing. Nat. Photonics.

[6] Bliokh KY, Bekshaev AY, Nori F (2013). Dual electromagnetism: Helicity, spin, momentum and angular momentum. New J. Phys..

[7] Allen L, Beijersbergen MW, Spreeuw RJC, Woerdman JP (1992). Orbital Angular-Momentum of Light and the Transformation of Laguerre-Gaussian Laser Modes. Phys. Rev. A.

[8] Bazhenov VY, Vasnetsov MV, Soskin MS (1990). Laser-Beams with Screw Dislocations in TheirWave-Fronts. JETP Lett..

[9] Meng X (2019). Generation of multiple beams carrying different orbital angular momentum modes based on anisotropic holographic metasurfaces in the radio-frequency domain. Applied Physics Letters.

[10] Heckenberg NR, McDuff R, Smith CP, White AG (1992). Generation of optical phase singularities by computer-generated holograms. Optics Letters.

[11] Tumbull, G. A., Robertson, D. A., Smith, G. M., Allen, L. & Padgett, M. J. The generation of free-space laguerre-gaussian modes at millimetre-wave frequencies by use of a spiral phaseplate Optical Angular Momentum, p. 186, (2003).

[12] Davis, J A *et al*. Review of operating modes for twisted nematic liquid crystal displays for applications in optical image processing. In Optical Science and Technology, SPIE’s 48th Annual Meeting, pp. 120–131. International Society for Optics and Photonics, (2003).

[13] Karimi E, Piccirillo B, Nagali E, Marrucci L, Santamato E (2009). Efficient generation and sorting of orbital angular momentum eigenmodes of light by thermally tuned q-plates. Applied Physics Letters.

[14] Gao X (2014). An orbital angular momentum radio communication system optimized by intensity-controlled masks effectively: Theoretical design and experimental verification. Applied Physics Letters.

[15] Gong Y (2017). Generation and transmission of OAM-carrying vortex beams using circular antenna array. IEEE Transactions on Antennas and Propagation.

[16] Ren J, Leung KW (2018). Generation of microwave orbital angular momentum states using hemispherical dielectric resonator antenna. Applied Physics Letters.

[17] Yu S (2016). Design, fabrication, and measurement of reflective metasurface for orbital angular momentum vortex wave in radio frequency domain. Applied Physics Letters.

[18] Meng X (2019). Generation of multiple beams carrying different orbital angular momentum modes based on anisotropic holographic metasurfaces in the radio-frequency domain. Applied Physics Letters.

[19] Zhang K (2019). High efficiency metalenses with switchable functionalities in microwave region. Applied Materials & Interfaces.

[20] Devlin RC (2017). Spin-to-orbital angular momentum conversion in dielectric metasurfaces. Opt. Express.

[21] Yang J (2018). Generation of radio vortex beams with designable polarization using anisotropic frequency selective surface. Applied Physics Letters.

[22] Karimipour M, Komjani N, Aryanian I (2019). Shaping electromagnetic Waves with flexible and continuous control of the Beam Directions Using Holography and convolution theorem. Scientific reports.

[23] Wang S-Y, Liu W, Geyi W (2018). Dual-band transmission polarization converter based on planar-dipole pair frequency selective surface. Sci. reports.

[24] Nayeri P, Yang F, Elsherbeni AZ (2012). Design and experiment of a single-feed quad-beam reflectarray antenna. IEEE Trans. Antennas Propag..

[25] Tremain B, Rance HJ, Hibbins AP, Sambles JR (2015). Polarization conversion from a thin cavity array in the microwave regime. Sci. reports.

[26] Georgi P, Schlickriede C, Li GX, Zhang S, Zentgraf T (2017). Rotational Doppler shift induced by spin-orbit coupling of light at spinning metasurfaces. Optica.

[27] Bouchard F (2014). Optical spin-to-orbital angular momentum conversion in ultra-thin metasurfaces with arbitrary topological charges. Appl. Phys. Lett..

[28] Chen MLN, Jiang LJ, Sha WEI (2016). Artificial perfect electric conductor-perfect magnetic conductor anisotropic metasurface for generating orbital angular momentum of microwave with nearly perfect conversion efficiency. J. Appl. Phys..

[29] Chen SM, Cai Y, Li GX, Zhang S, Cheah KW (2016). Geometric metasurface fork gratings for vortex-beam generation and manipulation. Laser Photonics Rev..

[30] Oliner A, Hessel A (1959). Guided waves on sinusoidally-modulated reactance surfaces. IRE Transactions on Antennas and Propagation.

[31] Moeini MM, Oraizi H, Amini A, Nayyeri V (2019). Wide-band beam-scanning by surface wave confinement on leaky wave holograms. Scientific reports.

[32] Monticone F, Alù A (2015). Leaky-wave theory, techniques, and applications: From microwaves to visible frequencies. Proceedings of the IEEE.

[33] Fong BH, Colburn JS, Ottusch JJ, Visher JL, Sievenpiper DF (2010). Scalar and tensor holographic artificial impedance surfaces. IEEE Transactions on Antennas and Propagation.

[34] Patel AM, Grbic A (2011). A printed leaky-wave antenna based on a sinusoidally-modulated reactance surface. IEEE Transactions on Antennas and Propagation.

[35] Gabor D (1948). A new microscopic principle. Nature.

[36] Checcacci PF, Russo V, Scheggi AM (1968). Holographic antennas. Proceedings of the IEEE.

[37] Li, Y. B., Cai, B. G., Cheng, Q. & Cui, T. J. Isotropic holographic metasurfaces for dual-functional radiations without mutual interferences. *Advanced Functional Materials* **26**, 29–35, 10.1002/adfm.201503654.

[38] Minatti G, Caminita F, Martini E, Sabbadini M, Maci S (2016). Synthesis of modulated-metasurface antennas with amplitude, phase, and polarization control. IEEE Transactions on Antennas and Propagation.

[39] Liu D, Cheng B, Pan X, Qiao L (2016). A horn-fed frequency scanning holographic antenna based on generalized law of reflection. Sci. reports.

[40] Hariharan, P. Basics of interferometry. 1 (Elsevier, 2007).

[41] Chen W (2017). Generation of wavelength-independent subwavelength Bessel beams using metasurfaces. Light: Science & Applications.

[42] Elliot, R. Antenna Theory and Design, Revised Ed (Wiley India Pvt. Limited, 2006).

[43] Li M, Xiao SQ, Sievenpiper DF (2016). Polarization-insensitive holographic surfaces with broadside radiation. IEEE Transactions on Antennas and Propagation.

[44] Oraizi, H., Amini, A., Abdolali, A. & Karimimehr, M. Design of wideband leaky wave antenna using sinusoidally modulated impedance surface based on the holography theory. IEEE Antennas and Wireless Propagation Letters 1–1, 10.1109/LAWP.2018.2866712 (2018).

[45] Pandi S, Balanis CA, Birtcher CR (2016). Analysis of wideband multilayered sinusoidally modulated metasurface. IEEE Antennas and Wireless Propagation Letters.

[46] Minatti G (2015). Modulated metasurface antennas for space: Synthesis, analysis and realizations. IEEE Transactions on Antennas and Propagation.

[47] Balanis, C. A. Antenna Theory: Analysis and Design. (Wiley, Inc., 4th ed., 2016).

